# Supplementary material for: Protocol for a scoping review of implementation research approaches to universal health coverage in Africa
Source: BMJ Open. 2021 Feb 15;11(2):e041721. doi: 10.1136/bmjopen-2020-041721 (PMC7887369; doi:10.1136/bmjopen-2020-041721)
Supplement: Supplementary data [file bmjopen-2020-041721supp001.pdf]

Appendix 1: Provisional PubMed/MEDLINE search strategy

Search #	Search Texts and Syntaxes
#1	"implementation science"[Title/Abstract] OR "implementation research"[Title/Abstract] OR "decision science"[Title/Abstract] OR "decision research"[Title/Abstract] OR "improvement science"[Title/Abstract] OR "improvement research"[Title/Abstract] OR "dissemination science"[Title/Abstract] OR "dissemination research"[Title/Abstract]
#2	"programme evaluation"[Title/Abstract] OR "outcome evaluation"[Title/Abstract] OR “process evaluation"[Title/Abstract] OR “impact evaluation"[Title/Abstract] OR “implementation evaluation"[Title/Abstract] OR “implementation fidelity”[Title/Abstract]
#3	facilitators[Title/Abstract] OR barriers[Title/Abstract] OR constraints [Title/Abstract] OR “implementation success”[Title/Abstract] OR implementation failure[Title/Abstract]
#4	#2 OR #3
#5	“Universal health coverage”[Title/Abstract] OR “Universal coverage”[Title/Abstract] OR “population coverage”[Title/Abstract] OR "health equity”[Title/Abstract] OR equity[Title/Abstract] OR equitability[Title/Abstract] OR Health[Title/Abstract] OR “health access”[Title/Abstract] OR “health services”[Title/Abstract] OR “health services accessibility”[Title/Abstract] OR access[Title/Abstract] OR accessibility[Title/Abstract] OR “health insurance"[Title/Abstract] OR “health care insurance"[Title/Abstract] OR “medical insurance"[Title/Abstract] OR “financial risk protection"[Title/Abstract] OR “out of pocket payment"[Title/Abstract] OR “out of pocket expenditure"[Title/Abstract] OR “out of pocket spending"[Title/Abstract]
#6	Africa OR African OR Algeria OR Angola OR Benin OR Botswana OR Burkina Faso OR Burundi OR Cameroon OR “Cape Verde” OR “Central African Republic” OR Chad OR Comoros OR Congo OR “Democratic Republic of Congo” OR Djibouti OR Egypt OR Eritrea OR Eswatini OR Ethiopia OR Gabon OR Gambia OR Ghana OR Guinea OR “Ivory Coast” OR “Cote d'Ivoire” OR Jamahiriya OR Kenya OR Lesotho OR Liberia OR Libya OR Madagascar OR Malawi OR Mali OR Mauritania OR Mauritius OR Mayotte OR Morocco OR Mozambique OR Namibia OR Niger OR Nigeria OR Principe OR Reunion OR Rwanda OR “Sao Tome” OR Senegal OR Seychelles OR “Sierra Leone” OR Somalia OR “St Helena” OR “sub-Saharan

	Africa" OR Sudan OR Swaziland OR Tanzania OR Togo OR Tunisia OR Uganda OR "Western Sahara" OR Zaire OR Zambia OR Zimbabwe
#7	#1 AND #4 AND #5 AND #6
