# Supplementary material for: Protocol for a scoping review of implementation research approaches to universal health coverage in Africa
Source: BMJ Open. 2021 Feb 15;11(2):e041721. doi: 10.1136/bmjopen-2020-041721 (PMC7887369; doi:10.1136/bmjopen-2020-041721)
Supplement: Author's manuscript [file bmjopen-2020-041721.draft_revisions.pdf]

BMJ Open is committed to open peer review. As part of this commitment we make the peer review history of every article we publish publicly available.

When an article is published we post the peer reviewers' comments and the authors' responses online. We also post the versions of the paper that were used during peer review. These are the versions that the peer review comments apply to.

The versions of the paper that follow are the versions that were submitted during the peer review process. They are not the versions of record or the final published versions. They should not be cited or distributed as the published version of this manuscript.

BMJ Open is an open access journal and the full, final, typeset and author-corrected version of record of the manuscript is available on our site with no access controls, subscription charges or pay-per-view fees (http://bmjopen.bmj.com).

If you have any questions on BMJ Open's open peer review process please email info.bmjopen@bmj.com

BMJ Open

A protocol for a scoping review of implementation research approaches for promoting universal health coverage in Africa

Journal:	*BMJ Open*
Manuscript ID	bmjopen-2020-041721
Article Type:	Protocol
Date Submitted by the Author:	17-Jun-2020
Complete List of Authors:	Nnaji, Chukwudi; University of Cape Town, School of Public Health and Family Medicine; South African Medical Research Council, Cochrane South Africa<br>Wiysonge, Charles; South African Medical Research Council, Cochrane South Africa; University of Cape Town, School of Public Health and Family Medicine<br>Okeibunor , J; World Health Organization Regional Office for Africa<br>Malinga, Thobile; South African Medical Research Council, Cochrane South Africa<br>Adamu, Abdu ; South African Medical Research Council, Cochrane South Africa ; Stellenbosch University, Centre for Evidence-based Health Care, Division of Epidemiology and Biostatistics, Department of Global Health, Faculty of Medicine and Health Sciences<br>Tumusiime, Prosper ; World Health Organization Regional Office for Africa<br>Karamagi, Humphrey ; World Health Organization Regional Office for Africa
Keywords:	Health policy < HEALTH SERVICES ADMINISTRATION & MANAGEMENT, PUBLIC HEALTH, Health economics < HEALTH SERVICES ADMINISTRATION & MANAGEMENT, Quality in health care < HEALTH SERVICES ADMINISTRATION & MANAGEMENT

SCHOLARONE™
Manuscripts

I, the Submitting Author has the right to grant and does grant on behalf of all authors of the Work (as defined in the below author licence), an exclusive licence and/or a non-exclusive licence for contributions from authors who are: i) UK Crown employees; ii) where BMJ has agreed a CC-BY licence shall apply, and/or iii) in accordance with the terms applicable for US Federal Government officers or employees acting as part of their official duties; on a worldwide, perpetual, irrevocable, royalty-free basis to BMJ Publishing Group Ltd ("BMJ") its licensees and where the relevant Journal is co-owned by BMJ to the co-owners of the Journal, to publish the Work in this journal and any other BMJ products and to exploit all rights, as set out in our licence.

The Submitting Author accepts and understands that any supply made under these terms is made by BMJ to the Submitting Author unless you are acting as an employee on behalf of your employer or a postgraduate student of an affiliated institution which is paying any applicable article publishing charge ("APC") for Open Access articles. Where the Submitting Author wishes to make the Work available on an Open Access basis (and intends to pay the relevant APC), the terms of reuse of such Open Access shall be governed by a Creative Commons licence – details of these licences and which Creative Commons licence will apply to this Work are set out in our licence referred to above.

Other than as permitted in any relevant BMJ Author's Self Archiving Policies, I confirm this Work has not been accepted for publication elsewhere, is not being considered for publication elsewhere and does not duplicate material already published. I confirm all authors consent to publication of this Work and authorise the granting of this licence.

For peer review only

A protocol for a scoping review of implementation research approaches for promoting universal health coverage in Africa

Chukwudi A Nnaji,[1,2] Charles S Wiysonge,[1,2,3] Joseph C Okeibunor,[4] Thobile Malinga,[1] Abdu A Adamu,[1,3] Prosper Tumusiime[4], Humphrey Karamagi,[4]

[1] Cochrane South Africa, South African Medical Research Council, Cape Town, South Africa

[2] School of Public Health and Family Medicine, University of Cape Town, Cape Town, South Africa

[3] Department of Global Health, Stellenbosch University, Cape Town, South Africa

[4] World Health Organization Regional Office for Africa, Brazzaville, Congo

Correspondence to Dr Chukwudi Nnaji; nnjchu001@myuct.ac.za

Word count: 3139

For peer review only

Abstract

Introduction: Implementation research has emerged as part of evidence-based decision-making efforts to plug current gaps in the translation of research evidence into health policy and practice. While there has been a growing number of institutions and initiatives promoting the uptake of implementation research in Africa, their role and effectiveness remain unclear, particularly in the context of universal health coverage (UHC). This review aims to extensively identify and characterise implementation research initiatives for promoting UHC in Africa.

Methods and analysis: This scoping review will be developed based on the methodological framework proposed by Arksey and O'Malley and enhanced by the Joanna Briggs Institute. It will be reported in accordance with the Preferred Reporting Items for Systematic reviews and Meta-Analyses extension for Scoping Reviews (PRISMA-ScR) guidelines. A comprehensive search of the following electronic databases will be conducted: MEDLINE (via PubMed), Scopus and the Cochrane Library. Relevant grey literature and reference lists will also be searched. All publications describing the application of implementation research in the context of UHC will be considered for inclusion. Findings will be narratively synthesized and analysed using a predefined conceptual framework. Where applicable, quantitative evidence will be aggregated using summary statistics. There will be consultation of stakeholders, including UHC-oriented health professionals, programme managers, implementation researchers and policy makers; to provide methodological, conceptual and practical insights.

Ethics and dissemination: The data used in this review will be sourced from publicly available literature, hence this study will not require ethical approval. Findings and recommendations will be disseminated to reach a diverse audience, including UHC advocates, implementation researchers and key health system stakeholders within the African region. Additionally, findings will be disseminated through an open-access publication in a relevant peer-reviewed journal.

Keywords: Implementation research, evidence, universal health coverage, access, equity

For peer review only

Strengths and limitations of the study

- This scoping review will be conducted in accordance with an enhanced evidence synthesis methodology and will use a well-grounded conceptual framework to map the evidence on implementation research in the context of UHC.
- It will contribute to filling an existing gap in the evidence relating to the relationship between implementation research and UHC-related outcomes.
- Multiple databases will be searched with a comprehensive search strategy to identify both peer-reviewed and relevant grey literature, with no language or document type restrictions.
- Broad stakeholder consultation with implementation researchers, UHC experts, health policy makers and programme managers in Africa will be incorporated into this scoping review to enhance conceptual and practical insights.
- Due to the broad nature of the topic, it is possible that some relevant literature may not be identified by our search strategy, however comprehensive.

For peer review only

Introduction

The need for health decision making to be informed by empirical evidence has been identified as a vital step for achieving universal health coverage (UHC) and equitable access to quality health care.[1,2] It has been recognised that decisions informed by research evidence have the potential to promote equitable service delivery and improve health outcomes at population level, while strengthening health systems.[2] The World Health Organization (WHO) defines UHC as "ensuring that all people have access to needed health services (including prevention, promotion, treatment, rehabilitation and palliation) of sufficient quality to be effective while also ensuring that the use of these services does not expose the user the financial hardship".[3] Since the 1978 Alma-Ata Declaration and the 1986 Ottawa Charter for Health Promotion, the right to the highest attainable standard of physical and mental health has gained increasing attention.[4] As a result of this prioritisation, UHC was adopted as a target of the Sustainable Development Goals (SDG), with the aspiration that countries will achieve this by 2030.[5]

With the increasing momentum of global efforts towards the attainment of UHC, countries are often faced with difficult choices regarding the most effective use of available health resources, particularly in contexts of resource limitation, competing healthcare needs and political priorities.[6] Given this inherent complexity, UHC decision making requires adequate consideration of best available and contextually applicable research evidence.[6,7] While investment in health research and research outputs have grown considerably in Africa over the years, there remain enormous gaps in translating available research evidence into health policy and practice.[8] This so-called 'know–do gap' has resulted in suboptimal gains from allocated health resources, in spite of growing investment towards the actualisation of UHC in Africa.[2,9] The gap is accentuated by the region's high burden of communicable and non-communicable diseases.[10,11]

Implementation science has emerged in response to this critical gap.[12] Implementation science is an integral part of the broader Evidence-informed Decision Making (EIDM) enterprise. EIDM involves processes of distilling and disseminating the best available evidence from research, practice and experience and using that evidence to inform and improve public health policy and practice.[13,14] Knowledge translation, knowledge transfer and translational research are EIDM

For peer review only

concepts that are closely related to implementation science, used to refer to the processes of moving research-based evidence into policy and practice, through the synthesis, dissemination, exchange and application of knowledge to improve the health of the population.[13,15-17] Although there may be nuanced differences in their conceptualisation, these terms essentially have similar goals and practical implications for improving health outconmes.[15-17]

There has been no clear consensus on the definition of implementation science.[18] In 2015, Odeny and colleagues published a review of the literature that found 73 unique definitions.[19] Broadly, implementation science has been defined as "the scientific study of methods to promote the systematic uptake of research findings and other evidence-based practices into routine practice, and, hence, to improve the quality and effectiveness of health services."[16] Since the field of implementation science has cogent applications for both clinical and public health settings, this definition is more encompassing and highlights the field's broad nature. The process of inquiry in implementation science is through research, which builds on traditional scientific methods, but focuses on a unique set of questions to improve the use of research in implementation.[16,19] Thus, implementation science offers the toolkit for addressing the know-do gap.[16,20,21]

Implementation research is an emerging sub-domain of implementation science that has been more distinctively defined. In 2006, Eccles and Mittman proposed a working definition for the field of implementation research – defining it as the "scientific study of methods to promote the adoption and integration of evidence based practices, interventions and policies into routine health care and public health settings."[21] More recently in 2013, the World Health Organization's Alliance for Health Policy and Systems Research (AHPSR) defines it as "the scientific study of the processes used in the implementation of initiatives as well as the contextual factors that affect these processes."[18] This definition highlights a defining feature of implementation research; that is, going beyond the study of methods of promoting the uptake of evidence into routine practice, to studying the contextual facilitators and barriers to evidence-based implementation.[17,18] For this reason, implementation research has been regarded as the heart and soul of implementation science.[17] While implementation science and implementation research have been interchangeably used in literature, implementation research will be the reference term for this review.

For peer review only

Various conceptual theories and frameworks have been used to guide implementation research efforts across diverse settings. Some of the most commonly used frameworks include the Consolidated Framework for Implementation Research (CFIR), Theoretical Domains Frameworks (TDF), Diffusion of Innovations (DI), Reach Effectiveness Adoption Implementation Maintenance (RE-AIM), Quality Implementation Framework (QIF), Interactive Systems Framework (ISF) and Normalisation Process Model (NPM).[22,23] Additionally, the use of adapted forms or combination of these frameworks has been reported.[22] To facilitate the use of implementation research in health system decision making and routine practice, there have to be: (a) availability of rigorous, robust, relevant, and reliable evidence, (b) decision-makers' appreciation of the value and importance of empirical evidence in decision making processes (c) a trusting, mutually respectful and enduring engagement between evidence producers and decision makers.[6,13,24]

Various implementation research initiatives and efforts for improving health outcomes have emerged in the African region in the last decade.[13,17,25-28] In spite of this substantial growth, implementation research uptake, effectiveness and scale-up in the region is challenged by numerous barriers.[25-27] These include inadequate research funding, limited availability and access to good quality research and paucity of contextually relevant evidence.[27] Other reported barriers include the untimeliness of research output and, of course, fragile collaboration between researchers and users of evidence like policy-makers and frontline programme implementers.[2,7,29,30]

Study rationale

Globally, evidence-based health decision making and implementation models are being adopted as approaches for improving the health of populations.[7,16,31] While there has been a growing number of institutions and initiatives promoting the uptake of implementation science and implementation research in Africa, the role and effectiveness of these initiatives remain unclear.[32,33]

For peer review only

Synthesised bodies of evidence on the role of implementation research in Africa's health systems and the extent to which it has been used to promote UHC and health equity on the continent, are sparse. With limited funding and institutional research capacity to drive implementation research efforts in Africa, there is an urgent need to seek out cross-country learning opportunities that can bolster understanding of implementation research and broader EIDM strategies in the region.[11,34] A better understanding will be important to stimulate greater synergy and collaboration between evidence producers and users, while optimising the overall effectiveness of implementation research efforts and health systems strengthening in the region.

Scoping reviews represent an appropriate methodology for thematically reviewing large bodies of literature in order to generate an overview of existing knowledge and practice, as well as identifying existing evidence gaps.[35,36] Like full systematic reviews, scoping reviews employ methods that are transparent and reproducible, using pre-defined search strategies and inclusion criteria.[37,38] However, unlike systematic reviews which often target specific and narrow research questions, scoping reviews typically have a broader focus – including the nature, volume and characteristics of the literature in order to identify, describe and categorise available evidence on the topic of interest.[36-38] This scoping review will be valuable for filling existing gaps in the availability of synthesised evidence on implementation research in the context of UHC, health equity and health systems strengthening within the African region. Additionally, it will map the region's implementation strategies, major actors, reported outcomes, facilitators and barriers from a diverse body of literature. Ultimately, it seeks to provide a holistic and user-friendly evidence summary of implementation research and key issues in the region for researchers, policy-makers and implementers, while identifying lingering knowledge and practice gaps to inform future implementation research efforts.

Study objectives

For peer review only

The aim of this review is to extensively scope the literature to identify and characterise implementation research initiatives for promoting UHC and health systems strengthening in Africa.

Methods

Conceptual framework

This scoping review will use the World Health Organization's 'UHC coverage cube' conceptual framework to guide the synthesis of evidence from literature and for characterising the nature of implementation research initiatives in the context of UHC.[39] This framework uses a cube (see Figure 1) to depict the multidimensional nature and outcomes of UHC. The cube illustrates three core dimensions of conceptualising UHC, these are in terms of population coverage of health-related social security systems, financial protection, and access to quality health care according to need.[39,40] These dimensions provide an assessment framework for UHC-targeted interventions, reflecting how many (or what proportion of) people received various needed health services of sufficient quality, while being protected from undue financial risks.[39] Although the framework does not take into account specific contextual factors, it has been widely used globally for conceptualising UHC across diverse health systems and contexts.[40-42]

Study design

The design of this scoping review will be developed based on the Arksey and O'Malley scoping review methodology[43], as enhanced by the Joanna Briggs Institute (JBI).[44] The JBI's enhanced framework expands the six stages of Arksey and O'Malley into 9 distinct stages for undertaking a scoping review: (1) defining the research question; (2) developing the inclusion and exclusion criteria; (3) describing the search strategy; (4) searching for the evidence; (5) selecting the evidence; (6) extracting the evidence; (7) charting the evidence; (8) summarising and reporting the evidence and (9) consulting with relevant stakeholders.

For peer review only

Stage 1: Defining the research question

Through consultation with the research team and key stakeholders, the overall main research question was defined as: 'What are the nature and scope of implementation research initiatives for improving equitable access to quality promotive, preventive, curative, rehabilitative and palliative health services in Africa?' For the purpose of this review, implementation research has been defined within the broader frameworks of implementation science, knowledge translation and evidence informed decision making. Based on the primary research question, the following specific research questions were defined:

1. How can implementation research help ensure that all people receive quality promotive, preventive, curative, rehabilitative and palliative services they need without suffering financial hardship in the African Region?
2. How can implementation research increase the population covered with health services in the African Region?
3. How can implementation research facilitate the realization of resilience and sustainability in African health systems?
4. What are the contextual facilitators and barriers to the uptake and sustainability of implementation research for promoting UHC in Africa?

Stage 2: Developing the inclusion and exclusion criteria

Inclusion criteria

These will be defined based on the PCC (Population, Concept and Contexts) framework, proposed by Peters and colleagues.[45] This framework is more appropriate for scoping reviews, compared with the commonly used PICO (Population, Intervention, Comparator and Outcome) framework, as it allows for the consideration of publications that may not feature all of the four PICO elements (e.g. lacking an outcome or comparator/control). Eligible population will include evidence producers (health researchers), intermediaries (such as knowledge brokers and

For peer review only

implementation research institutions) and evidence users (such as health policymakers, programme implementers like non-government organisations and healthcare providers). There are two concepts of interest for this review, an intervention concept (implementation research) and an outcome concept (UHC). The two concepts of interest are implementation research and UHC. To be considered for inclusion, studies must report on UHC-related interventions or strategies that made use of specific implementation research frameworks. These may be any activity designed to facilitate the use of research-based knowledge in UHC-related decision making (including policymaking, programme implementation and frontline service-delivery decision making). Studies with or without comparison between implementation research strategies and controls will be eligible for inclusion. UHC outcomes will include health service coverage, access (service utilisation and quality of care) and financial risk protection, in line with the Cube framework.[39] Studies that evaluated specific health programme implementation outcomes, barriers or facilitators, will be included, provided the implementation involved the use of specific implementation research approaches, frameworks or models. Context will be health systems in Africa. Any type of primary study design will be eligible, including randomised controlled trials and observational studies.

Exclusion criteria

Literature focused solely or mainly on theoretical and conceptual development of implementation research will be excluded, as will those evaluating implementation research knowledge and perception, those evaluating implementation outcomes without using specific implementation research approaches and those reporting implementation research outcomes that are not UHC-related. Multinational literature involving African and non-African countries and meeting inclusion criteria will be excluded if country-specific information cannot be abstracted.

Stage 3: Describing the search strategy

The search strategy will be developed with the guidance of a reference librarian, and adapted for other databases using appropriate controlled vocabulary and syntax. The search strategy will use

For peer review only

search terms that are sensitive enough to capture literature relevant to implementation research, with due cognisance of the field's diverse and overlapping nomenclature and search filters for African countries. An initial exploration of current available literature on implementation research and UHC will help guide the selection of search terms, ensuring they are inclusive enough to capture any UHC-related implementation research intervention. The search strategy will be applied in accordance with the Peer Review of Electronic Search Strategies (PRESS) guidelines.[46] A provisional MEDLINE search strategy is illustrated in Appendix 1.

For peer review only

Stage 4: Searching the evidence

A comprehensive literature search will be conducted on the following electronic databases: MEDLINE (via PubMed), Scopus and Cochrane Library (including the Cochrane Central Register of Controlled Trials (CENTRAL) and the Database of Abstracts of Reviews of Effects (DARE)). Each database will be searched from inception to the date of search. Additionally, relevant grey literature will be searched for implementation research-related reports, including the website of the WHO Alliance for Health Policy and Systems Research (AHPSR). Websites of known implementation research institutions, networks and collaborations will be explored. We will also conduct a hand-search of reference lists of relevant literature to identify for potentially eligible literature. No language restriction will be applied. If a potentially eligible literature was published in a language other than English, a language translation will be sought.

Stage 5: Selecting the evidence

The review process will consist of two levels of screening: a title and abstract screening to identify potentially eligible publications and review of full texts to select those to be included in the review based on pre-defined inclusion/exclusion criteria. The first level will involve the independent screening of titles and abstracts of all retrieved citations from the search output by CAN and TM. Articles that are deemed relevant will be included in the full-text review. Following

the removal of duplicates, full texts of remaining studies will be retrieved. In the second step, the retrieved full texts will be assessed in duplicate by CAN and TM to determine if they meet the inclusion/exclusion criteria. Those meeting the inclusion criteria will be included in the review. Discrepancies in study selection between the two independent reviewer will be discussed to reach a consensus. Where a consensus is not reached, a third reviewer (CSW) will arbitrate.

Stage 6: Extracting the evidence

A data extraction tool (using a Microsoft Excel spreadsheet) will be developed by the research team to extract relevant info from included literature. Information to be extracted will include at least the following:

1. Author(s).

2. Year of publication.

3. Country where the evidence/study was published/conducted.

4. Aims/purpose.

5. Study population and study size.

6. Type of evidence/study design

7. Implementation research strategy type and comparator (if applicable).

8. Duration of intervention.

9. Universal health coverage outcomes reported (e.g. population coverage, access and financial risk protection).

10. Key findings that relate to scoping review objectives.

Other categories that come up during the data extraction process will be discussed by the research team and added to the data extraction tool. The tool will be reviewed by the research team and pretested before use. Data abstraction will be conducted in duplicate by two

For peer review only

independent reviewers. To ensure accurate data collection, each reviewer's independently abstracted data will be compared, and any discordance will be resolved through a consensus. Where a consensus is not reached after discussion between the two independent reviewers, a third reviewer will arbitrate. All collected data will be collated in a single Microsoft Excel spreadsheet for validation and coding.

Stage 7: Charting the evidence

The data extraction table produced will include the description of each included evidence/study using the 10 information headings described in Stage 5 above, including other categories that may come up during the data extraction process. To ensure accuracy of charted evidence, each reviewer's independent charted data will be compared and any discrepancies will be iteratively discussed by the researchers to ensure consistency between the reviewers.

Stage 8: Summarising and reporting the evidence

Findings of the review will be reported using the Preferred Reporting Items for Systematic reviews and Meta-Analyses extension for Scoping Reviews (PRISMA-ScR) checklist.[47] Findings will be summarised and reported using narrative descriptions based on themes that will emerge from the charted evidence. The results will be compared and consolidated through consensus between the two reviewers. Where applicable, quantitative evidence will be aggregated using summary statistics. As the purpose of a scoping review is to aggregate evidence and present a summary of the evidence rather than to evaluate the quality of the individual evidence, this review's overall assessment of the strength of the synthesised evidence will be narrative rather than quantitative.

Stage 9: Consultation

Consultations will provide opportunities for stakeholder involvement, providing additional insights beyond what is reported in the literature.[48] Given the potentially diverse nature of

For peer review only

implementation research literature, a broad array of stakeholders will be consulted, from implementation researchers to UHC-oriented health professionals, programme managers and policy makers. These stakeholders can help to identify grey literature that may not be obtainable from scholarly database searches, as well as providing methodological, conceptual and practical insights for guiding the interpretation of findings.

Patient and public involvement

Patients and the public were not involved in the development of this protocol.

Ethics and dissemination

Since the scoping review methodology involves reviewing and collecting data from publicly available materials, this study will not require ethics approval. To facilitate dissemination of findings, the research team will use a multi-stakeholder approach in presenting the findings to key health system stakeholders within the African region, in addition to open-access publication in a relevant peer-reviewed journal.

Authors' contributions

The study was conceived by CSW, JCO, and HK. CAN wrote the first draft of the manuscript with guidance from CSW. CSW, JCO, TM, AAA, PT, and HK contributed to writing the final version of the manuscript. All the authors read and approved the final manuscript.

Acknowledgements

The authors gratefully acknowledge the valuable comments and inputs from the following colleagues at Cochrane South Africa: Chinwe Iwu-Jaja, Selvan Naidoo, Phelele Njenje, Jill Ryan, and Alison Wiyeh.

For peer review only

Funding

The WHO Regional Office for Africa and the South African Medical Research Council (SAMRC), through Cochrane South Africa, provided funding for this study. The views expressed are those of the authors and do not necessarily reflect those of WHO, the SAMRC, Cochrane, or any other organisation that the authors are affiliated with.

Competing interests

None declared

Patient and public involvement

Not required

For peer review only

References

1. Conalogue DM, Kinn S, Mulligan J-A, et al.: International consultation on long-term global health research priorities, research capacity and research uptake in developing countries. Health research policy and systems 2017; 15:24-.
2. Edwards A, Zweigenthal V, Olivier J: Evidence map of knowledge translation strategies, outcomes, facilitators and barriers in African health systems. Health Research Policy and Systems 2019; 17:16.
3. Mathur MR, Williams DM, Reddy KS, et al.: Universal health coverage: a unique policy opportunity for oral health. J Dent Res 2015; 94:3S-5S.
4. Puras D: Universal Health Coverage: A Return to Alma-Ata and Ottawa. Health Hum Rights 2016; 18:7-10.
5. Hogan DR, Stevens GA, Hosseinpoor AR, et al.: Monitoring universal health coverage within the Sustainable Development Goals: development and baseline data for an index of essential health services. The Lancet Global Health 2018; 6:e152-e68.
6. Jessani NS, Siddiqi SM, Babcock C, et al.: Factors affecting engagement between academic faculty and decision-makers: learnings and priorities for a school of public health. Health Research Policy and Systems 2018; 16:65.
7. Brownson RC, Fielding JE, Green LW: Building Capacity for Evidence-Based Public Health: Reconciling the Pulls of Practice and the Push of Research. Annu Rev Public Health 2018; 39:27-53.
8. Grimshaw JM, Eccles MP, Lavis JN, et al.: Knowledge translation of research findings. Implement Sci 2012; 7:50-.
9. Tetroe JM, Graham ID, Foy R, et al.: Health research funding agencies' support and promotion of knowledge translation: an international study. Milbank Q 2008; 86:125-55.
10. Poot CC, van der Kleij RM, Brakema EA, et al.: From research to evidence-informed decision making: a systematic approach. Journal of Public Health 2018; 40:i3-i12.

11. Shroff Z, Aulakh B, Gilson L, et al.: Incorporating research evidence into decision-making processes: researcher and decision-maker perceptions from five low- and middle-income countries. Health research policy and systems 2015; 13:70-.
12. National Academies of Sciences, Engineering, and Medicine. 2016. Applying an Implementation Science Approach to Genomic Medicine: Workshop Summary. Washington, DC: The National Academies Press. https://doi.org/10.17226/23403.
13. Jessani NS, Hendricks L, Nicol L, et al.: University Curricula in Evidence-Informed Decision Making and Knowledge Translation: Integrating Best Practice, Innovation, and Experience for Effective Teaching and Learning. Front Public Health 2019; 7:313-.
14. Yousefi-Nooraie R, Dobbins M, Brouwers M, et al.: Information seeking for making evidence-informed decisions: a social network analysis on the staff of a public health department in Canada. BMC Health Serv Res 2012; 12:118-.
15. Graham ID, Logan J, Harrison MB, et al.: Lost in knowledge translation: Time for a map? Journal of Continuing Education in the Health Professions 2006; 26:13-24.
16. Bauer MS, Damschroder L, Hagedorn H, et al.: An introduction to implementation science for the non-specialist. BMC Psychol 2015; 3:32-.
17. Hoke T. Implementation research: The unambiguous cornerstone of implementation science. Research for evidence. FHI 360 Research. Available via: https://researchforevidence.fhi360.org/implementation-research-the-unambiguous-cornerstone-of-implementation-science Accessed on 27 December 2019s.
18. David H. Peters, Nhan T. Tran, Taghreed Adam. Implementation research in health: a practical guide. Alliance for Health Policy and Systems Research, World Health Organization, 2013. Available via: https://apps.who.int/iris/bitstream/handle/10665/91758/9789241506212_eng.pdf;jsessionid=A55D0E6349536138CC3C24C865C15991?sequence=1.
19. Odeny TA, Padian N, Doherty MC, et al.: Definitions of implementation science in HIV/AIDS. Lancet HIV 2015; 2:e178-e80.
20. Kao LS: Implementation Science and Quality Improvement. Success in Academic Surgery: Health Services Research Ed. Dimick JB, Greenberg CC. London, Springer London, 2014; 85-100.
21. Eccles MP, Mittman BS: Welcome to Implementation Science. Implementation Science 2006; 1:1.
22. Birken SA, Powell BJ, Shea CM, et al.: Criteria for selecting implementation science theories and frameworks: results from an international survey. Implementation Science 2017; 12:124.
23. Foy R, Ovretveit J, Shekelle PG, et al.: The role of theory in research to develop and evaluate the implementation of patient safety practices. BMJ Qual Saf 2011; 20:453-9.
24. Ross S, Lavis J, Rodriguez C, et al.: Partnership experiences: involving decision-makers in the research process. J Health Serv Res Policy 2003; 8 Suppl 2:26-34.
25. Oliver K, Innvar S, Lorenc T, et al.: A systematic review of barriers to and facilitators of the use of evidence by policymakers. BMC Health Serv Res 2014; 14:2-.
26. Oliver TR: The politics of public health policy. Annu Rev Public Health 2006; 27:195-233.
27. Ongolo-Zogo P, Lavis JN, Tomson G, et al.: Initiatives supporting evidence informed health system policymaking in Cameroon and Uganda: a comparative historical case study. BMC Health Serv Res 2014; 14:612.
28. World Health Organization (WHO). Supporting the Use of Research Evidence (SURE) for Policy in African Health Systems. Avialable via: https://www.who.int/evidence/partners/SURE.pdf Accessed on 12 Janunary 2020.
29. Langlois EV, Becerril Montekio V, Young T, et al.: Enhancing evidence informed policymaking in complex health systems: lessons from multi-site collaborative approaches. Health research policy and systems 2016; 14:20-.

For peer review only

30. Kothari A, Wathen CN: Integrated knowledge translation: digging deeper, moving forward. Journal of epidemiology and community health 2017; 71:619-23.
31. Vasan A, Mabey DC, Chaudhri S, et al.: Support and performance improvement for primary health care workers in low- and middle-income countries: a scoping review of intervention design and methods. Health Policy Plan 2017; 32:437-52.
32. Tricco AC, Zarin W, Rios P, et al.: Barriers, facilitators, strategies and outcomes to engaging policymakers, healthcare managers and policy analysts in knowledge synthesis: a scoping review protocol. BMJ Open 2016; 6:e013929-e.
33. Uzochukwu B, Onwujekwe O, Mbachu C, et al.: The challenge of bridging the gap between researchers and policy makers: experiences of a Health Policy Research Group in engaging policy makers to support evidence informed policy making in Nigeria. Globalization and Health 2016; 12:67.
34. Kirigia JM, Pannenborg CO, Amore LGC, et al.: Global Forum 2015 dialogue on "From evidence to policy - thinking outside the box": perspectives to improve evidence uptake and good practices in the African Region. BMC Health Serv Res 2016; 16 Suppl 4:215-.
35. Bussiek P-BV, De Poli C, Bevan G: A scoping review protocol to map the evidence on interventions to prevent overweight and obesity in children. BMJ Open 2018; 8:e019311-e.
36. Wickremasinghe D, Kuruvilla S, Mays N, et al.: Taking knowledge users' knowledge needs into account in health: an evidence synthesis framework. Health Policy Plan 2016; 31:527-37.
37. Bragge P, Clavisi O, Turner T, et al.: The Global Evidence Mapping Initiative: scoping research in broad topic areas. BMC Med Res Methodol 2011; 11:92-.
38. Munn Z, Peters MDJ, Stern C, et al.: Systematic review or scoping review? Guidance for authors when choosing between a systematic or scoping review approach. BMC Med Res Methodol 2018; 18:143-.
39. World Health Organization (WHO). The universal health coverage (UHC) cube. Health financing for universal coverage and health system performance: concepts and implications for policy. Available via: https://www.who.int/bulletin/volumes/91/8/12-113985/en/.
40. Abiiro GA, De Allegri M: Universal health coverage from multiple perspectives: a synthesis of conceptual literature and global debates. BMC Int Health Hum Rights 2015; 15:17.
41. Nygren-Krug H: The Right(s) Road to Universal Health Coverage. Health Hum Rights 2019; 21:215-28.
42. Obermann K, Chanturidze T, Glazinski B, et al.: The shaded side of the UHC cube: a systematic review of human resources for health management and administration in social health protection schemes. Health Econ Rev 2018; 8:4-.
43. Arksey H, O'Malley L: Scoping studies: towards a methodological framework. International Journal of Social Research Methodology 2005; 8:19-32.
44. Peters M, Godfrey C, McInerney P, et al. Scoping Reviews. In: Aromataris E MZE, ed. Joanna Briggs Institute reviewer's manual the Joanna Briggs Institute, 2017. Available via: https://wiki.joannabriggs.org/display/MANUAL/11.1.3+The+scoping+review+framework Accessed 16 February 2020.
45. Peters MDJ, Godfrey CM, Khalil H, et al.: Guidance for conducting systematic scoping reviews. Int J Evid Based Healthc 2015; 13:141-6.
46. McGowan J, Sampson M, Salzwedel DM, et al.: PRESS Peer Review of Electronic Search Strategies: 2015 Guideline Statement. J Clin Epidemiol 2016; 75:40-6.
47. Tricco AC, Lillie E, Zarin W, et al.: PRISMA Extension for Scoping Reviews (PRISMA-ScR): Checklist and Explanation. Annals of internal medicine 2018; 169:467-73.
48. Levac D, Colquhoun H, O'Brien KK: Scoping studies: advancing the methodology. Implement Sci 2010; 5:69-.

For peer review only

Supplementary files

Figure 1: The World Health Organization UHC Cube

Appendix 1: Provisional PubMed/MEDLINE search strategy

For peer review only

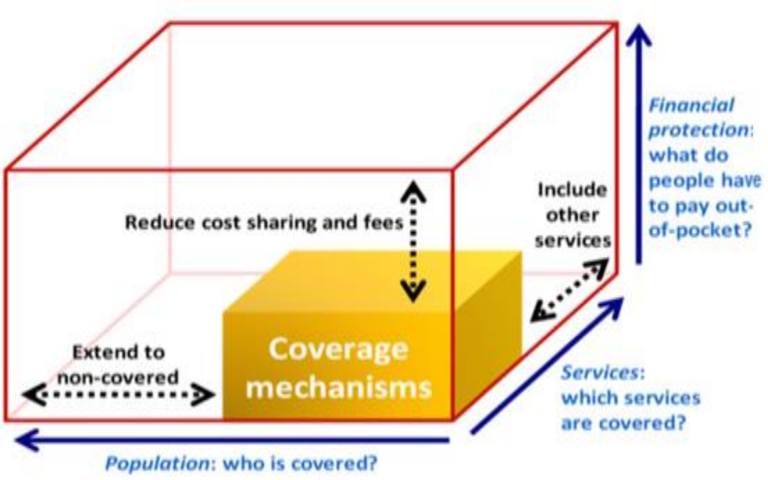

Figure 1: The World Health Organization's UHC Cube[1]

1. World Health Organization (WHO). Health financing for universal coverage. Available via: https://www.who.int/health_financing/topics/benefit-package/UHC-choices-facing-purchasers/en/.

Appendix 1: Provisional PubMed/MEDLINE search strategy

Search #	Search Texts and Syntaxes
#1	"implementation science"[Title/Abstract] OR "implementation research"[Title/Abstract] OR "decision science"[Title/Abstract] OR "decision research"[Title/Abstract] OR "improvement science"[Title/Abstract] OR "improvement research"[Title/Abstract]
#2	"knowledge translation"[Title/Abstract] OR "knowledge management"[Title/Abstract] OR "dissemination science"[Title/Abstract] OR "dissemination research"[Title/Abstract]
#3	"evidence-based medicine"[MeSH Terms] OR "evidence based medicine"[Title/Abstract] OR "evidence based healthcare"[Title/Abstract] OR "evidence based health care"[Title/Abstract] OR "evidence informed decision making"[Title/Abstract]
#4	#1 OR #2 OR #3
#5	"Universal health coverage"[Title/Abstract] OR "health equity"[Title/Abstract] OR Health[Title/Abstract] OR "health access"[Title/Abstract] OR "financial risk protection"[Title/Abstract] OR "health access"[Title/Abstract] OR access[Title/Abstract] OR equity[Title/Abstract]
#6	Africa OR African OR Algeria OR Angola OR Benin OR Botswana OR Burkina Faso OR Burundi OR Cameroon OR "Canary Islands" OR "Cape Verde" OR "Central African Republic" OR Chad OR Comoros OR Congo OR "Democratic Republic of Congo" OR Djibouti OR Egypt OR Eritrea OR Eswatini OR Ethiopia OR Gabon OR Gambia OR Ghana OR Guinea OR "Ivory Coast" OR "Cote d'Ivoire" OR Jamahiriya OR Kenya OR Lesotho OR Liberia OR Libya OR Madagascar OR Malawi OR Mali OR Mauritania OR Mauritius OR Mayotte OR Morocco OR Mozambique OR Namibia OR Niger OR Nigeria OR Principe OR Reunion OR Rwanda OR "Sao Tome" OR Senegal OR Seychelles OR "Sierra Leone" OR Somalia OR "St Helena" OR "sub-Saharan Africa" OR Sudan OR Swaziland OR Tanzania OR Togo OR Tunisia OR Uganda OR "Western Sahara" OR Zaire OR Zambia OR Zimbabwe
#7	#4 AND #5 AND #6

For peer review only

PRISMA-P (Preferred Reporting Items for Systematic review and Meta-Analysis Protocols) 2015 checklist: recommended items to address in a systematic review protocol*

Section and topic	Item No	Checklist item	Page
**ADMINISTRATIVE INFORMATION**			
Title:			1
Identification	1a	Identify the report as a protocol of a systematic review	
Update	1b	If the protocol is for an update of a previous systematic review, identify as such	
Registration	2	If registered, provide the name of the registry (such as PROSPERO) and registration number	N/A
Authors:			
Contact	3a	Provide name, institutional affiliation, e-mail address of all protocol authors; provide physical mailing address of corresponding author	1
Contributions	3b	Describe contributions of protocol authors and identify the guarantor of the review	
Amendments	4	If the protocol represents an amendment of a previously completed or published protocol, identify as such and list changes; otherwise, state plan for documenting important protocol amendments	N/A
Support:			
Sources	5a	Indicate sources of financial or other support for the review	
Sponsor	5b	Provide name for the review funder and/or sponsor	15
Role of sponsor or funder	5c	Describe roles of funder(s), sponsor(s), and/or institution(s), if any, in developing the protocol	
**INTRODUCTION**			
Rationale	6	Describe the rationale for the review in the context of what is already known	6
Objectives	7	Provide an explicit statement of the question(s) the review will address with reference to participants, interventions, comparators, and outcomes (PICO)	7
**METHODS**			
Eligibility criteria	8	Specify the study characteristics (such as PICO, study design, setting, time frame) and report characteristics (such as years considered, language, publication status) to be used as criteria for eligibility for the review	9
Information sources	9	Describe all intended information sources (such as electronic databases, contact with study authors, trial registers or other grey literature sources) with planned dates of coverage	11
Search strategy	10	Present draft of search strategy to be used for at least one electronic database, including planned limits, such that it could be repeated	11
Study records:			
Data management	11a	Describe the mechanism(s) that will be used to manage records and data throughout the review	

For peer review only

Selection process	11b	State the process that will be used for selecting studies (such as two independent reviewers) through each phase of the review (that is, screening, eligibility and inclusion in meta-analysis)	
Data collection process	11c	Describe planned method of extracting data from reports (such as piloting forms, done independently, in duplicate), any processes for obtaining and confirming data from investigators	12
Data items	12	List and define all variables for which data will be sought (such as PICO items, funding sources), any pre-planned data assumptions and simplifications	
Outcomes and prioritization	13	List and define all outcomes for which data will be sought, including prioritization of main and additional outcomes, with rationale	12
Risk of bias in individual studies	14	Describe anticipated methods for assessing risk of bias of individual studies, including whether this will be done at the outcome or study level, or both; state how this information will be used in data synthesis	N/A
Data synthesis	15a	Describe criteria under which study data will be quantitatively synthesised	13
	15b	If data are appropriate for quantitative synthesis, describe planned summary measures, methods of handling data and methods of combining data from studies, including any planned exploration of consistency (such as I^2, Kendall's τ)	
	15c	Describe any proposed additional analyses (such as sensitivity or subgroup analyses, meta-regression)	
	15d	If quantitative synthesis is not appropriate, describe the type of summary planned	
Meta-bias(es)	16	Specify any planned assessment of meta-bias(es) (such as publication bias across studies, selective reporting within studies)	N/A
Confidence in cumulative evidence	17	Describe how the strength of the body of evidence will be assessed (such as GRADE)	N/A

*** It is strongly recommended that this checklist be read in conjunction with the PRISMA-P Explanation and Elaboration (cite when available) for important clarification on the items. Amendments to a review protocol should be tracked and dated.** **The copyright for PRISMA-P (including checklist) is held by the PRISMA-P Group and is distributed under a Creative Commons Attribution Licence 4.0.**

From: Shamseer L, Moher D, Clarke M, Ghersi D, Liberati A, Petticrew M, Shekelle P, Stewart L, PRISMA-P Group. Preferred reporting items for systematic review and meta-analysis protocols (PRISMA-P) 2015: elaboration and explanation. BMJ. 2015 Jan 2;349(jan02 1):g7647.

For peer review only

BMJ Open

A protocol for a scoping review of implementation research approaches to universal health coverage in Africa

Journal:	*BMJ Open*
Manuscript ID	bmjopen-2020-041721.R1
Article Type:	Protocol
Date Submitted by the Author:	07-Nov-2020
Complete List of Authors:	Nnaji, Chukwudi; University of Cape Town, School of Public Health and Family Medicine; South African Medical Research Council, Cochrane South Africa<br>Wiysonge, Charles; South African Medical Research Council, Cochrane South Africa; University of Cape Town, School of Public Health and Family Medicine<br>Okeibunor , J; World Health Organization Regional Office for Africa<br>Malinga, Thobile; South African Medical Research Council, Cochrane South Africa<br>Adamu, Abdu ; South African Medical Research Council, Cochrane South Africa ; Stellenbosch University, Centre for Evidence-based Health Care, Division of Epidemiology and Biostatistics, Department of Global Health, Faculty of Medicine and Health Sciences<br>Tumusiime, Prosper ; World Health Organization Regional Office for Africa<br>Karamagi, Humphrey ; World Health Organization Regional Office for Africa
**Primary Subject Heading**:	Public health
Secondary Subject Heading:	Public health, Evidence based practice, Global health, Health services research, Research methods
Keywords:	Health policy < HEALTH SERVICES ADMINISTRATION & MANAGEMENT, PUBLIC HEALTH, Health economics < HEALTH SERVICES ADMINISTRATION & MANAGEMENT, Quality in health care < HEALTH SERVICES ADMINISTRATION & MANAGEMENT
Note: The following files were submitted by the author for peer review, but cannot be converted to PDF. You must view these files (e.g. movies) online.	
Main Document.docx	

SCHOLARONE™
Manuscripts

I, the Submitting Author has the right to grant and does grant on behalf of all authors of the Work (as defined in the below author licence), an exclusive licence and/or a non-exclusive licence for contributions from authors who are: i) UK Crown employees; ii) where BMJ has agreed a CC-BY licence shall apply, and/or iii) in accordance with the terms applicable for US Federal Government officers or employees acting as part of their official duties; on a worldwide, perpetual, irrevocable, royalty-free basis to BMJ Publishing Group Ltd ("BMJ") its licensees and where the relevant Journal is co-owned by BMJ to the co-owners of the Journal, to publish the Work in this journal and any other BMJ products and to exploit all rights, as set out in our licence.

The Submitting Author accepts and understands that any supply made under these terms is made by BMJ to the Submitting Author unless you are acting as an employee on behalf of your employer or a postgraduate student of an affiliated institution which is paying any applicable article publishing charge ("APC") for Open Access articles. Where the Submitting Author wishes to make the Work available on an Open Access basis (and intends to pay the relevant APC), the terms of reuse of such Open Access shall be governed by a Creative Commons licence – details of these licences and which Creative Commons licence will apply to this Work are set out in our licence referred to above.

Other than as permitted in any relevant BMJ Author's Self Archiving Policies, I confirm this Work has not been accepted for publication elsewhere, is not being considered for publication elsewhere and does not duplicate material already published. I confirm all authors consent to publication of this Work and authorise the granting of this licence.

For peer review only

Appendix 1: Provisional PubMed/MEDLINE search strategy

Search #	Search Texts and Syntaxes
#1	"implementation science"[Title/Abstract] OR "implementation research"[Title/Abstract] OR "decision science"[Title/Abstract] OR "decision research"[Title/Abstract] OR "improvement science"[Title/Abstract] OR "improvement research"[Title/Abstract] OR "dissemination science"[Title/Abstract] OR "dissemination research"[Title/Abstract]
#2	"knowledge translation"[Title/Abstract] OR "knowledge management"[Title/Abstract] OR "know-do gap""[Title/Abstract] OR "knowledge transfer"[Title/Abstract] OR knowledge-to-action[Title/Abstract]
#3	"evidence-based healthcare"[Title/Abstract] OR "evidence-based health care"[Title/Abstract] OR "evidence-informed decision making"[Title/Abstract] OR "evidence-informed healthcare decision making"[Title/Abstract]
#4	#1 OR #2 OR #3
#5	"Universal health coverage"[Title/Abstract] OR "Universal coverage"[Title/Abstract] OR "population coverage"[Title/Abstract] OR "health equity"[Title/Abstract] OR equity[Title/Abstract] OR equitability[Title/Abstract] OR Health[Title/Abstract] OR "health access"[Title/Abstract] OR "health services"[Title/Abstract] OR "health services accessibility"[Title/Abstract] OR access[Title/Abstract] OR accessibility[Title/Abstract] OR "health insurance"[Title/Abstract] OR "health care insurance"[Title/Abstract] OR "medical insurance"[Title/Abstract] OR "financial risk protection"[Title/Abstract] OR "out of pocket payment"[Title/Abstract] OR "out of pocket expenditure"[Title/Abstract] OR "out of pocket spending"[Title/Abstract]
#6	Africa OR African OR Algeria OR Angola OR Benin OR Botswana OR Burkina Faso OR Burundi OR Cameroon OR "Cape Verde" OR "Central African Republic" OR Chad OR Comoros OR Congo OR "Democratic Republic of Congo" OR Djibouti OR Egypt OR Eritrea OR Eswatini OR Ethiopia OR Gabon OR Gambia OR Ghana OR Guinea OR "Ivory Coast" OR "Cote d'Ivoire" OR Jamahiriya OR Kenya OR Lesotho OR Liberia OR Libya OR Madagascar OR Malawi OR Mali OR Mauritania OR Mauritius OR Mayotte OR Morocco OR Mozambique OR Namibia OR Niger OR Nigeria OR Principe OR Reunion OR Rwanda OR "Sao Tome" OR Senegal OR Seychelles OR "Sierra Leone" OR Somalia OR "St Helena" OR "sub-Saharan

For peer review only

	Africa" OR Sudan OR Swaziland OR Tanzania OR Togo OR Tunisia OR Uganda OR "Western Sahara" OR Zaire OR Zambia OR Zimbabwe
#7	#4 AND #5 AND #6

For peer review only

Appendix 1

Preferred Reporting Items for Systematic reviews and Meta-Analyses extension for Scoping Reviews (PRISMA-ScR) Checklist

SECTION	ITEM	PRISMA-ScR CHECKLIST ITEM	REPORTED ON PAGE #
**TITLE**			
Title	1	Identify the report as a scoping review.	1
**ABSTRACT**			
Structured summary	2	Provide a structured summary that includes (as applicable): background, objectives, eligibility criteria, sources of evidence, charting methods, results, and conclusions that relate to the review questions and objectives.	2
**INTRODUCTION**			
Rationale	3	Describe the rationale for the review in the context of what is already known. Explain why the review questions/objectives lend themselves to a scoping review approach.	6-7
Objectives	4	Provide an explicit statement of the questions and objectives being addressed with reference to their key elements (e.g., population or participants, concepts, and context) or other relevant key elements used to conceptualize the review questions and/or objectives.	7
**METHODS**			
Protocol and registration	5	Indicate whether a review protocol exists; state if and where it can be accessed (e.g., a Web address); and if available, provide registration information, including the registration number.	n/a
Eligibility criteria	6	Specify characteristics of the sources of evidence used as eligibility criteria (e.g., years considered, language, and publication status), and provide a rationale.	9-10
Information sources*	7	Describe all information sources in the search (e.g., databases with dates of coverage and contact with authors to identify additional sources), as well as the date the most recent search was executed.	10-11
Search	8	Present the full electronic search strategy for at least 1 database, including any limits used, such that it could be repeated.	Appendix 1
Selection of sources of evidence†	9	State the process for selecting sources of evidence (i.e., screening and eligibility) included in the scoping review.	11
Data charting process‡	10	Describe the methods of charting data from the included sources of evidence (e.g., calibrated forms or forms that have been tested by the team before their use, and whether data charting was done independently or in duplicate) and any processes for obtaining and confirming data from investigators.	12
Data items	11	List and define all variables for which data were sought and any assumptions and simplifications made.	11-12
Critical appraisal of individual sources of evidence§	12	If done, provide a rationale for conducting a critical appraisal of included sources of evidence; describe the methods used and how this information was used in any data synthesis (if appropriate).	n/a

For peer review only - http://bmjopen.bmj.com/site/about/guidelines.xhtml

SECTION	ITEM	PRISMA-ScR CHECKLIST ITEM	REPORTED ON PAGE #
Synthesis of results	13	Describe the methods of handling and summarizing the data that were charted.	13
**RESULTS**			
Selection of sources of evidence	14	Give numbers of sources of evidence screened, assessed for eligibility, and included in the review, with reasons for exclusions at each stage, ideally using a flow diagram.	n/a
Characteristics of sources of evidence	15	For each source of evidence, present characteristics for which data were charted and provide the citations.	n/a
Critical appraisal within sources of evidence	16	If done, present data on critical appraisal of included sources of evidence (see item 12).	n/a
Results of individual sources of evidence	17	For each included source of evidence, present the relevant data that were charted that relate to the review questions and objectives.	n/a
Synthesis of results	18	Summarize and/or present the charting results as they relate to the review questions and objectives.	n/a
**DISCUSSION**			
Summary of evidence	19	Summarize the main results (including an overview of concepts, themes, and types of evidence available), link to the review questions and objectives, and consider the relevance to key groups.	n/a
Limitations	20	Discuss the limitations of the scoping review process.	n/a
Conclusions	21	Provide a general interpretation of the results with respect to the review questions and objectives, as well as potential implications and/or next steps.	n/a
**FUNDING**			
Funding	22	Describe sources of funding for the included sources of evidence, as well as sources of funding for the scoping review. Describe the role of the funders of the scoping review.	14

JBI = Joanna Briggs Institute; PRISMA-ScR = Preferred Reporting Items for Systematic reviews and Meta-Analyses extension for Scoping Reviews.
* Where *sources of evidence* (see second footnote) are compiled from, such as bibliographic databases, social media platforms, and Web sites.
† A more inclusive/heterogeneous term used to account for the different types of evidence or data sources (e.g., quantitative and/or qualitative research, expert opinion, and policy documents) that may be eligible in a scoping review as opposed to only studies. This is not to be confused with *information sources* (see first footnote).
‡ The frameworks by Arksey and O'Malley (6) and Levac and colleagues (7) and the JBI guidance (4, 5) refer to the process of data extraction in a scoping review as data charting.
§ The process of systematically examining research evidence to assess its validity, results, and relevance before using it to inform a decision. This term is used for items 12 and 19 instead of "risk of bias" (which is more applicable to systematic reviews of interventions) to include and acknowledge the various sources of evidence that may be used in a scoping review (e.g., quantitative and/or qualitative research, expert opinion, and policy document).

Adapted from: Tricco AC, Lillie E, Zarin W, O'Brien KK, Colquhoun H, Levac D, et al. PRISMA Extension for Scoping Reviews (PRISMAScR): Checklist and Explanation. Ann Intern Med. 2018;169:467–473. doi: 10.7326/M18-0850.

For peer review only

A protocol for a scoping review of implementation research approaches to universal health coverage in Africa

Journal:	*BMJ Open*
Manuscript ID	bmjopen-2020-041721.R2
Article Type:	Protocol
Date Submitted by the Author:	08-Jan-2021

Complete List of Authors:	Nnaji, Chukwudi; University of Cape Town, School of Public Health and Family Medicine; South African Medical Research Council, Cochrane South Africa<br>Wiysonge, Charles; South African Medical Research Council, Cochrane South Africa; University of Cape Town, School of Public Health and Family Medicine<br>Okeibunor , J; World Health Organization Regional Office for Africa<br>Malinga, Thobile; South African Medical Research Council, Cochrane South Africa<br>Adamu, Abdu ; South African Medical Research Council, Cochrane South Africa ; Stellenbosch University, Centre for Evidence-based Health Care, Division of Epidemiology and Biostatistics, Department of Global Health, Faculty of Medicine and Health Sciences<br>Tumusiime, Prosper ; World Health Organization Regional Office for Africa<br>Karamagi, Humphrey ; World Health Organization Regional Office for Africa

**Primary Subject Heading**:	Public health
Secondary Subject Heading:	Public health, Evidence based practice, Global health, Health services research, Research methods
Keywords:	Health policy < HEALTH SERVICES ADMINISTRATION & MANAGEMENT, PUBLIC HEALTH, Health economics < HEALTH SERVICES ADMINISTRATION & MANAGEMENT, Quality in health care < HEALTH SERVICES ADMINISTRATION & MANAGEMENT

SCHOLARONE™
Manuscripts

I, the Submitting Author has the right to grant and does grant on behalf of all authors of the Work (as defined in the below author licence), an exclusive licence and/or a non-exclusive licence for contributions from authors who are: i) UK Crown employees; ii) where BMJ has agreed a CC-BY licence shall apply, and/or iii) in accordance with the terms applicable for US Federal Government officers or employees acting as part of their official duties; on a worldwide, perpetual, irrevocable, royalty-free basis to BMJ Publishing Group Ltd ("BMJ") its licensees and where the relevant Journal is co-owned by BMJ to the co-owners of the Journal, to publish the Work in this journal and any other BMJ products and to exploit all rights, as set out in our licence.

The Submitting Author accepts and understands that any supply made under these terms is made by BMJ to the Submitting Author unless you are acting as an employee on behalf of your employer or a postgraduate student of an affiliated institution which is paying any applicable article publishing charge ("APC") for Open Access articles. Where the Submitting Author wishes to make the Work available on an Open Access basis (and intends to pay the relevant APC), the terms of reuse of such Open Access shall be governed by a Creative Commons licence – details of these licences and which Creative Commons licence will apply to this Work are set out in our licence referred to above.

Other than as permitted in any relevant BMJ Author's Self Archiving Policies, I confirm this Work has not been accepted for publication elsewhere, is not being considered for publication elsewhere and does not duplicate material already published. I confirm all authors consent to publication of this Work and authorise the granting of this licence.

For peer review only

A protocol for a scoping review of implementation research approaches to universal health coverage in Africa

Chukwudi A Nnaji,[1,2] Charles S Wiysonge,[1,2,3] Joseph C Okeibunor,[4] Thobile Malinga,[1] Abdu A Adamu,[1,3] Prosper Tumusiime[4], Humphrey Karamagi,[4]

[1] Cochrane South Africa, South African Medical Research Council, Cape Town, South Africa

[2] School of Public Health and Family Medicine, University of Cape Town, Cape Town, South Africa

[3] Department of Global Health, Stellenbosch University, Cape Town, South Africa

[4] World Health Organization Regional Office for Africa, Brazzaville, Congo

Correspondence to Dr Chukwudi Nnaji; nnjchu001@myuct.ac.za

Word count: 3172

For peer review only

Abstract

Introduction: Implementation research has emerged as part of evidence-based decision-making efforts to plug current gaps in the translation of research evidence into health policy and practice. While there has been a growing number of institutions and initiatives promoting the uptake of implementation research in Africa, their role and effectiveness remain unclear, particularly in the context of universal health coverage (UHC). This review aims to extensively identify and characterise the nature, facilitators and barriers to the use of implementation research for assessing or evaluating UHC-related interventions or programmes in Africa.

Methods and analysis: This scoping review will be developed based on the methodological framework proposed by Arksey and O'Malley and enhanced by the Joanna Briggs Institute. It will be reported in accordance with the Preferred Reporting Items for Systematic reviews and Meta-Analyses extension for Scoping Reviews (PRISMA-ScR) guidelines. A comprehensive search of the following electronic databases will be conducted: MEDLINE (via PubMed), Scopus and the Cochrane Library. Relevant grey literature and reference lists will also be searched. All publications describing the application of implementation research in the context of UHC will be considered for inclusion. Findings will be narratively synthesized and analysed using a predefined conceptual framework. Where applicable, quantitative evidence will be aggregated using summary statistics. There will be consultation of stakeholders, including UHC-oriented health professionals, programme managers, implementation researchers and policy makers; to provide methodological, conceptual and practical insights.

Ethics and dissemination: The data used in this review will be sourced from publicly available literature, hence this study will not require ethical approval. Findings and recommendations will be disseminated to reach a diverse audience, including UHC advocates, implementation researchers and key health system stakeholders within the African region. Additionally, findings will be disseminated through an open-access publication in a relevant peer-reviewed journal.

Keywords: Implementation research, evidence, universal health coverage, access, equity

For peer review only

For peer review only - http://bmjopen.bmj.com/site/about/guidelines.xhtml

Strengths and limitations of the study

- This scoping review will be conducted in accordance with an enhanced evidence synthesis methodology.
- It will use a well-grounded conceptual framework to map the evidence on implementation research in the context of UHC.
- Multiple databases will be searched with a comprehensive search strategy to identify both peer-reviewed and relevant grey literature sources.
- Broad consultation with stakeholders will be incorporated to enhance the review's conceptual and methodological rigour.
- Due to the broad nature of the topic, it is possible that some relevant literature may not be identified by our search strategy, however comprehensive.

For peer review only

Introduction

The need for health decision making to be informed by empirical evidence has been identified as a vital step for achieving universal health coverage (UHC) and equitable access to quality health care.[1,2] It has been recognised that decisions informed by research evidence have the potential to promote equitable access to health services and improve health outcomes at the population level, while strengthening health systems.[2] The World Health Organization (WHO) defines UHC as “ensuring that all people have access to needed health services (including prevention, promotion, treatment, rehabilitation and palliation) of sufficient quality to be effective while also ensuring that the use of these services does not expose the user the financial hardship”.[3] Since the 1978 Alma-Ata Declaration and the 1986 Ottawa Charter for Health Promotion, the right to the highest attainable standard of physical and mental health has gained increasing attention.[4] As a result of this prioritisation, UHC was adopted as a target of the Sustainable Development Goals (SDG), with the aspiration that countries will achieve this by 2030.[5]

With the increasing momentum of global efforts towards the attainment of UHC, countries are often faced with difficult choices regarding the most effective use of available health resources, particularly in contexts of resource limitation, competing healthcare needs and political priorities.[6] Given this inherent complexity, UHC decision making requires adequate consideration of best available and contextually applicable research evidence.[6,7] While investment in health research and research outputs have grown considerably in Africa over the years, there remain enormous gaps in translating available research evidence into health policy and practice.[8] This so-called ‘know–do gap’ has resulted in suboptimal gains from allocated health resources, in spite of growing investment towards the actualisation of UHC in Africa.[2,9] The gap is accentuated by the region’s high burden of communicable and non-communicable diseases.[10,11]

Implementation science has emerged in response to this critical gap.[12] Implementation science is an integral part of the broader Evidence-informed Decision Making (EIDM) enterprise. EIDM involves processes of distilling and disseminating the best available evidence from research, practice and experience and using that evidence to inform and improve public health policy and practice.[13,14] Knowledge translation, knowledge transfer and translational research are EIDM

For peer review only - http://bmjopen.bmj.com/site/about/guidelines.xhtml

concepts that are closely related to implementation science, used to refer to the processes of moving research-based evidence into policy and practice, through the synthesis, dissemination, exchange and application of knowledge to improve the health of the population.[13,15-17] Although there may be nuanced differences in their conceptualisation, these terms essentially have similar goals and practical implications for improving health outconmes.[15-17]

There has been no clear consensus on the definition of implementation science.[18] In 2015, Odeny and colleagues published a review of the literature that found 73 unique definitions.[19] Broadly, implementation science has been defined as: "the scientific study of methods to promote the systematic uptake of research findings and other evidence-based practices into routine practice, and, hence, to improve the quality and effectiveness of health services."[16] Since the field of implementation science has cogent applications in both clinical and public health settings, this definition is more encompassing and underscores the field's broad nature. The process of inquiry in implementation science is through research, which builds on traditional scientific methods, but focuses on a unique set of questions to improve the use of research in implementation.[16,19] Thus, implementation science offers the toolkit for addressing the know-do gap.[16,20,21]

Implementation research is an emerging sub-domain of implementation science that has been more distinctively defined. In 2006, Eccles and Mittman proposed a working definition for the field of implementation research – defining it as the "scientific study of methods to promote the adoption and integration of evidence based practices, interventions and policies into routine health care and public health settings."[21] More recently in 2013, the World Health Organization's Alliance for Health Policy and Systems Research (AHPSR) defines it as "the scientific study of the processes used in the implementation of initiatives as well as the contextual factors that affect these processes."[18] This definition highlights a defining feature of implementation research; that is, going beyond the study of methods of promoting the uptake of evidence into routine practice, to studying the contextual facilitators and barriers to evidence-based implementation.[17,18] For this reason, implementation research has been regarded as the heart and soul of implementation science.[17] While implementation science and implementation research have been interchangeably used in literature, implementation research will be the reference term for this review.

For peer review only - http://bmjopen.bmj.com/site/about/guidelines.xhtml

Various conceptual theories and frameworks have been used to guide implementation research efforts across diverse settings. Some of the most commonly used frameworks include the Consolidated Framework for Implementation Research (CFIR), Theoretical Domains Frameworks (TDF), Diffusion of Innovations (DI), Reach Effectiveness Adoption Implementation Maintenance (RE-AIM), Quality Implementation Framework (QIF), Interactive Systems Framework (ISF) and Normalisation Process Model (NPM).[22,23] Additionally, the use of adapted forms or combination of these frameworks has been reported.[22] To facilitate the use of implementation research in health system decision making and routine practice, there have to be: (a) availability of rigorous, robust, relevant, and reliable evidence, (b) decision-makers' appreciation of the value and importance of empirical evidence in decision making processes (c) a trusting, mutually respectful and enduring engagement between evidence producers and decision makers.[6,13,24]

Various implementation research initiatives and efforts for evaluating and improving health programme outcomes have emerged in the African region in the last decade.[13,17,25-28] In spite of this substantial growth, implementation research uptake, effectiveness and scale-up in the region is challenged by numerous barriers.[25-27] These include inadequate research funding, limited availability and access to good quality research and paucity of contextually relevant evidence.[27] Other reported barriers include the untimeliness of research output and, of course, fragile collaboration between researchers and users of evidence like policy-makers and frontline programme implementers.[2,7,29,30]

Study rationale

Globally, evidence-based health decision making and implementation models are being adopted as approaches for improving the health of populations.[7,16,31] While there has been a growing number of institutions and initiatives promoting the uptake of implementation research in Africa, the role and effectiveness of these initiatives remain unclear, particularly in UHC contexts.[32,33]

Synthesised bodies of evidence on the role of implementation research in Africa's health systems and the extent to which it has been used to promote UHC and health equity on the continent,

For peer review only - http://bmjopen.bmj.com/site/about/guidelines.xhtml

are sparse. With limited funding and institutional research capacity to drive implementation research efforts in Africa, there is an urgent need to seek out cross-country learning opportunities that can bolster understanding of implementation research and broader EIDM strategies in the region.[11,34] A better understanding will be important to stimulate greater uptake, better application and sustainability of implementation research strategies within UHC contexts in the region.

Scoping reviews represent an appropriate methodology for thematically reviewing large bodies of literature in order to generate an overview of existing knowledge and practice, as well as identifying existing evidence gaps.[35,36] Like full systematic reviews, scoping reviews employ methods that are transparent and reproducible, using pre-defined search strategies and inclusion criteria.[37,38] However, unlike systematic reviews which often target specific and narrow research questions, scoping reviews typically have a broader focus – including the nature, volume and characteristics of the literature in order to identify, describe and categorise available evidence on the topic of interest.[36-38] This scoping review will be valuable for filling existing gaps in the availability of synthesised evidence on implementation research in the context of UHC, health equity and health systems strengthening within the African region. Additionally, it will map the region's implementation strategies, major actors, reported outcomes, facilitators and barriers from a diverse body of literature. Ultimately, it seeks to provide a holistic and user-friendly evidence summary of implementation research and key issues in the region for researchers, policy-makers and implementers, while identifying lingering knowledge and practice gaps to inform future implementation research efforts.

Study objectives

The aim of this review is to extensively scope the literature to identify and characterise the nature, facilitators and barriers to the use of implementation research for assessing or evaluating UHC-related interventions or programmes in the African region.

For peer review only - http://bmjopen.bmj.com/site/about/guidelines.xhtml

Methods

Conceptual framework

This scoping review will follow the implementation science taxonomy proposed by Ridde and colleagues[39] to guide the synthesis of identified evidence and characterising the nature of implementation research strategies in the context of UHC. To help characterise and describe the possible implementation research approaches, frameworks and theories, this taxonomy defines four models commonly used in implementation science (intervention theory, framework, middle-range theory and grand theory). These models form a continuum and may overlap when used. Implementation scientists and researchers use these models for three main implementation studies: fidelity assessment, process evaluation and complex evaluation.[39]

Study design

The design of this scoping review will be developed based on the Arksey and O'Malley scoping review methodology[40], as enhanced by the Joanna Briggs Institute (JBI).[41] The JBI's enhanced framework expands the six stages of Arksey and O'Malley into 9 distinct stages for undertaking a scoping review: (1) defining the research question; (2) developing the inclusion and exclusion criteria; (3) describing the search strategy; (4) searching for the evidence; (5) selecting the evidence; (6) extracting the evidence; (7) charting the evidence; (8) summarising and reporting the evidence and (9) consulting with relevant stakeholders.

Stage 1: Defining the research question

Through consultation with the research team and key stakeholders, the overall main research question was defined as: 'What are the nature, facilitators and barriers of implementation research strategies for promoting UHC in Africa?' For the purpose of this review, implementation research has been defined within the broader frameworks of implementation science,

For peer review only

For peer review only - http://bmjopen.bmj.com/site/about/guidelines.xhtml

knowledge translation and evidence informed decision making. Based on the primary research question, the following specific research questions were defined:

1. How has implementation research been used to assess or evaluate UHC-related interventions or programmes in the African Region?
2. What are the contextual facilitators and barriers to the application of implementation research in assessing or evaluating UHC-related interventions or programmes in Africa?

Stage 2: Developing the inclusion and exclusion criteria

Inclusion criteria

These will be defined based on the PCC (Population, Concept and Contexts) framework, proposed by Peters and colleagues.[42] This framework is more appropriate for scoping reviews, compared with the commonly used PICO (Population, Intervention, Comparator and Outcome) framework, as it allows for the consideration of publications that may not feature all of the four PICO elements (e.g. lacking an outcome or comparator/control). Eligible population will include evidence producers (health researchers), intermediaries (such as knowledge brokers and implementation research institutions) and evidence users (such as health policymakers, programme implementers like non-government organisations and healthcare providers). The concept of interest is implementation research. To be considered for inclusion, studies must report on the use of implementation research strategies, models, theories or frameworks for assessing or evaluating UHC-related interventions or programmes. These may include activities such as fidelity assessment, process evaluation, outcome evaluation or complex evaluation.[39] Studies with or without comparison between implementation research strategies and controls will be eligible for inclusion. UHC outcomes will include scope of package of health services; population coverage, access and service utilisation; quality of care; and financial risk protection, in line with the Cube framework.[43] Studies that evaluated specific health programme implementation outcomes, barriers or facilitators, will be included, provided the evaluation involved the use of specified implementation research approaches, frameworks or models.

For peer review only - http://bmjopen.bmj.com/site/about/guidelines.xhtml

Context will be health systems within the African region (Appendix 1 specifies the countries and territories of focus within the region). Any type of primary study design will be eligible, including randomised controlled trials and observational studies.

Exclusion criteria

Literature focused solely or mainly on theoretical and conceptual development of implementation research will be excluded; as will study protocols and studies evaluating implementation outcomes without specifying or mentioning the implementation research approaches, models, theories or frameworks used. Multinational literature involving African and non-African countries and meeting inclusion criteria will be excluded if country-specific information cannot be abstracted.

Stage 3: Describing the search strategy

The search strategy will be developed with the guidance of a reference librarian, and adapted for other databases using appropriate controlled vocabulary and syntax. The search strategy will use search terms that are sensitive enough to capture literature sources relevant to implementation research, with due cognisance of the field's diverse and overlapping nomenclature and search filters for African countries. An initial exploration of current available literature on implementation research and UHC will help guide the selection of search terms, ensuring they are inclusive enough to capture any UHC-related implementation research intervention. The search strategy will be applied in accordance with the Peer Review of Electronic Search Strategies (PRESS) guidelines.[44] A provisional MEDLINE search strategy is illustrated in Appendix 1.

Stage 4: Searching the evidence

A comprehensive literature search will be conducted on the following electronic databases: MEDLINE (via PubMed), Scopus and Cochrane Library (including the Cochrane Central Register of

For peer review only - http://bmjopen.bmj.com/site/about/guidelines.xhtml

Controlled Trials (CENTRAL) and the Database of Abstracts of Reviews of Effects (DARE)). Each database will be searched from the year 2000 (coinciding with the inception of implementation science as a field in the mid-2000s) to the date of search. Additionally, relevant grey literature will be searched for implementation research-related reports, including the website of the WHO Alliance for Health Policy and Systems Research (AHPSR). Websites of known implementation research institutions, networks and collaborations will be explored. We will also conduct hand-searches of reference lists of relevant literature to identify potentially eligible literature. Only literature sources published in English will be eligible for inclusion.

Stage 5: Selecting the evidence

The review process will consist of two levels of screening: a title and abstract screening to identify potentially eligible publications and review of full texts to select those to be included in the review based on pre-defined inclusion/exclusion criteria. The first level will involve the independent screening of titles and abstracts of all retrieved citations from the search output by CAN and TM. Articles that are deemed relevant will be included in the full-text review. Following the removal of duplicates, full texts of remaining studies will be retrieved. In the second step, the retrieved full texts will be assessed in duplicate by CAN and TM to determine if they meet the inclusion/exclusion criteria. Those meeting the inclusion criteria will be included in the review. Discrepancies in study selection between the two independent reviewer will be discussed to reach a consensus. Where a consensus is not reached, a third reviewer (CSW) will arbitrate.

Stage 6: Extracting the evidence

A data extraction tool (using a Microsoft Excel spreadsheet) will be developed by the research team to extract relevant info from included literature. Information to be extracted will include at least the following:

1. Author(s).

For peer review only - http://bmjopen.bmj.com/site/about/guidelines.xhtml

2. Year of publication.

3. Country where the evidence/study was published/conducted.

4. Aims/purpose.

5. Study population and study size.

6. Type of evidence/study design.

7. Implementation research strategy, model, theory or framework used

8. Duration of implementation research.

9. Type of UHC-related programme or intervention involved (classified by programmatic area of focus).

10. Key implementation research findings.

11. Reported implementation research facilitators and barriers.

Other categories that come up during the data extraction process will be discussed by the research team and added to the data extraction tool. The tool will be reviewed by the research team and pretested before use. Data abstraction will be conducted in duplicate by two independent reviewers. To ensure accurate data collection, each reviewer's independently abstracted data will be compared, and any discordance will be resolved through a consensus. Where a consensus is not reached after discussion between the two independent reviewers, a third reviewer will arbitrate. All collected data will be collated in a single Microsoft Excel spreadsheet for validation and coding.

Stage 7: Charting the evidence

A table describing each included study will be presented using the 11 information headings described in Stage 5 above. To ensure accuracy of charted evidence, each reviewer’s independent charted data will be compared and any discrepancies will be iteratively discussed by the researchers to ensure consistency between the reviewers.

For peer review only - http://bmjopen.bmj.com/site/about/guidelines.xhtml

Stage 8: Summarising and reporting the evidence

Findings of the review will be reported using the Preferred Reporting Items for Systematic reviews and Meta-Analyses extension for Scoping Reviews (PRISMA-ScR) checklist.[45] A PRISMA flow diagram will be used to illustrate the literature search results and study selection process. Findings will be summarised and reported using narrative descriptions based on the following themes: country-context, implementation research strategy used and type of UHC-related programme or intervention involved. The implementation science taxonomy proposed by Ridde and colleagues[39] will be used to classify identified implementation research models, theories or frameworks. Implementation research facilitators and barriers will be reported based on the themes that will emerge from the charted evidence. Where applicable, quantitative evidence will be aggregated using summary statistics. As the purpose of a scoping review is to aggregate evidence and present a summary of the evidence rather than to evaluate the quality of the individual evidence, this review will not involve any formal appraisal of the quality of included evidence.

Stage 9: Consultation

Multidisciplinary and multinational consultations will provide opportunities for stakeholders to provide additional insights beyond what is reported in the literature.[46] Given the potentially diverse nature of implementation research literature, a broad array of stakeholders will be consulted, from implementation researchers to UHC-oriented health professionals, programme managers and policy makers. These stakeholders can help to identify grey literature that may not be obtainable from scholarly database searches, as well as providing methodological, conceptual and practical insights for guiding the interpretation and dissemination of findings.

Patient and public involvement

For peer review only - http://bmjopen.bmj.com/site/about/guidelines.xhtml

Patients and the public were not involved in the development of this protocol.

Ethics and dissemination

Since the scoping review methodology involves reviewing and collecting data from publicly available materials, this study will not require ethics approval. To facilitate dissemination of findings, the research team will use a multi-stakeholder approach in presenting the findings to key health system stakeholders within the African region, in addition to open-access publication in a relevant peer-reviewed journal.

Authors' contributions

The study was conceived by CSW, JCO, and HK. CAN wrote the first draft of the manuscript with guidance from CSW. CSW, JCO, TM, AAA, PT, and HK contributed to writing the final version of the manuscript. All the authors read and approved the final manuscript.

Acknowledgements

The authors gratefully acknowledge the valuable comments and inputs from the following colleagues at Cochrane South Africa: Chinwe Iwu-Jaja, Selvan Naidoo, Phelele Njenje, Jill Ryan, and Alison Wiyeh.

Funding

The WHO Regional Office for Africa and the South African Medical Research Council (SAMRC), through Cochrane South Africa, provided funding for this study. The views expressed are those of the authors and do not necessarily reflect those of WHO, the SAMRC, Cochrane, or any other organisation that the authors are affiliated with.

Competing interests

None declared

For peer review only

References

1. Conalogue DM, Kinn S, Mulligan J-A, et al.: International consultation on long-term global health research priorities, research capacity and research uptake in developing countries. Health research policy and systems 2017; 15:24-.
2. Edwards A, Zweigenthal V, Olivier J: Evidence map of knowledge translation strategies, outcomes, facilitators and barriers in African health systems. Health Research Policy and Systems 2019; 17:16.
3. Mathur MR, Williams DM, Reddy KS, et al.: Universal health coverage: a unique policy opportunity for oral health. J Dent Res 2015; 94:3S-5S.
4. Puras D: Universal Health Coverage: A Return to Alma-Ata and Ottawa. Health Hum Rights 2016; 18:7-10.
5. Hogan DR, Stevens GA, Hosseinpoor AR, et al.: Monitoring universal health coverage within the Sustainable Development Goals: development and baseline data for an index of essential health services. The Lancet Global Health 2018; 6:e152-e68.
6. Jessani NS, Siddiqi SM, Babcock C, et al.: Factors affecting engagement between academic faculty and decision-makers: learnings and priorities for a school of public health. Health Research Policy and Systems 2018; 16:65.
7. Brownson RC, Fielding JE, Green LW: Building Capacity for Evidence-Based Public Health: Reconciling the Pulls of Practice and the Push of Research. Annu Rev Public Health 2018; 39:27-53.
8. Grimshaw JM, Eccles MP, Lavis JN, et al.: Knowledge translation of research findings. Implement Sci 2012; 7:50-.
9. Tetroe JM, Graham ID, Foy R, et al.: Health research funding agencies' support and promotion of knowledge translation: an international study. Milbank Q 2008; 86:125-55.
10. Poot CC, van der Kleij RM, Brakema EA, et al.: From research to evidence-informed decision making: a systematic approach. Journal of Public Health 2018; 40:i3-i12.
11. Shroff Z, Aulakh B, Gilson L, et al.: Incorporating research evidence into decision-making processes: researcher and decision-maker perceptions from five low- and middle-income countries. Health research policy and systems 2015; 13:70-.
12. National Academies of Sciences, Engineering, and Medicine. 2016. Applying an Implementation Science Approach to Genomic Medicine: Workshop Summary. Washington, DC: The National Academies Press. https://doi.org/10.17226/23403.
13. Jessani NS, Hendricks L, Nicol L, et al.: University Curricula in Evidence-Informed Decision Making and Knowledge Translation: Integrating Best Practice, Innovation, and Experience for Effective Teaching and Learning. Front Public Health 2019; 7:313-.
14. Yousefi-Nooraie R, Dobbins M, Brouwers M, et al.: Information seeking for making evidence-informed decisions: a social network analysis on the staff of a public health department in Canada. BMC Health Serv Res 2012; 12:118-.
15. Graham ID, Logan J, Harrison MB, et al.: Lost in knowledge translation: Time for a map? Journal of Continuing Education in the Health Professions 2006; 26:13-24.
16. Bauer MS, Damschroder L, Hagedorn H, et al.: An introduction to implementation science for the non-specialist. BMC Psychol 2015; 3:32-.
17. Hoke T. Implementation research: The unambiguous cornerstone of implementation science. Research for evidence. FHI 360 Research. Available via:

For peer review only - http://bmjopen.bmj.com/site/about/guidelines.xhtml

https://researchforevidence.fhi360.org/implementation-research-the-unambiguous-cornerstone-of-implementation-science Accessed on 27 December 2019s.

18. David H. Peters, Nhan T. Tran, Taghreed Adam. Implementation research in health: a practical guide. Alliance for Health Policy and Systems Research, World Health Organization, 2013. Available via: https://apps.who.int/iris/bitstream/handle/10665/91758/9789241506212_eng.pdf;jsessionid=A55D0E6349536138CC3C24C865C15991?sequence=1.
19. Odeny TA, Padian N, Doherty MC, et al.: Definitions of implementation science in HIV/AIDS. Lancet HIV 2015; 2:e178-e80.
20. Kao LS: Implementation Science and Quality Improvement. Success in Academic Surgery: Health Services Research Ed. Dimick JB, Greenberg CC. London, Springer London, 2014; 85-100.
21. Eccles MP, Mittman BS: Welcome to Implementation Science. Implementation Science 2006; 1:1.
22. Birken SA, Powell BJ, Shea CM, et al.: Criteria for selecting implementation science theories and frameworks: results from an international survey. Implementation Science 2017; 12:124.
23. Foy R, Ovretveit J, Shekelle PG, et al.: The role of theory in research to develop and evaluate the implementation of patient safety practices. BMJ Qual Saf 2011; 20:453-9.
24. Ross S, Lavis J, Rodriguez C, et al.: Partnership experiences: involving decision-makers in the research process. J Health Serv Res Policy 2003; 8 Suppl 2:26-34.
25. Oliver K, Innvar S, Lorenc T, et al.: A systematic review of barriers to and facilitators of the use of evidence by policymakers. BMC Health Serv Res 2014; 14:2-.
26. Oliver TR: The politics of public health policy. Annu Rev Public Health 2006; 27:195-233.
27. Ongolo-Zogo P, Lavis JN, Tomson G, et al.: Initiatives supporting evidence informed health system policymaking in Cameroon and Uganda: a comparative historical case study. BMC Health Serv Res 2014; 14:612.
28. World Health Organization (WHO). Supporting the Use of Research Evidence (SURE) for Policy in African Health Systems. Avialable via: https://www.who.int/evidence/partners/SURE.pdf Accessed on 12 Janunary 2020.
29. Langlois EV, Becerril Montekio V, Young T, et al.: Enhancing evidence informed policymaking in complex health systems: lessons from multi-site collaborative approaches. Health research policy and systems 2016; 14:20-.
30. Kothari A, Wathen CN: Integrated knowledge translation: digging deeper, moving forward. Journal of epidemiology and community health 2017; 71:619-23.
31. Vasan A, Mabey DC, Chaudhri S, et al.: Support and performance improvement for primary health care workers in low- and middle-income countries: a scoping review of intervention design and methods. Health Policy Plan 2017; 32:437-52.
32. Tricco AC, Zarin W, Rios P, et al.: Barriers, facilitators, strategies and outcomes to engaging policymakers, healthcare managers and policy analysts in knowledge synthesis: a scoping review protocol. BMJ Open 2016; 6:e013929-e.
33. Uzochukwu B, Onwujekwe O, Mbachu C, et al.: The challenge of bridging the gap between researchers and policy makers: experiences of a Health Policy Research Group in engaging policy makers to support evidence informed policy making in Nigeria. Globalization and Health 2016; 12:67.
34. Kirigia JM, Pannenborg CO, Amore LGC, et al.: Global Forum 2015 dialogue on "From evidence to policy - thinking outside the box": perspectives to improve evidence uptake and good practices in the African Region. BMC Health Serv Res 2016; 16 Suppl 4:215-.
35. Bussiek P-BV, De Poli C, Bevan G: A scoping review protocol to map the evidence on interventions to prevent overweight and obesity in children. BMJ Open 2018; 8:e019311-e.

For peer review only

For peer review only - http://bmjopen.bmj.com/site/about/guidelines.xhtml

36. Wickremasinghe D, Kuruvilla S, Mays N, et al.: Taking knowledge users' knowledge needs into account in health: an evidence synthesis framework. Health Policy Plan 2016; 31:527-37.
37. Bragge P, Clavisi O, Turner T, et al.: The Global Evidence Mapping Initiative: scoping research in broad topic areas. BMC Med Res Methodol 2011; 11:92-.
38. Munn Z, Peters MDJ, Stern C, et al.: Systematic review or scoping review? Guidance for authors when choosing between a systematic or scoping review approach. BMC Med Res Methodol 2018; 18:143-.
39. Ridde V, Pérez D, Robert E: Using implementation science theories and frameworks in global health. BMJ global health 2020; 5:e002269.
40. Arksey H, O'Malley L: Scoping studies: towards a methodological framework. International Journal of Social Research Methodology 2005; 8:19-32.
41. Peters M, Godfrey C, McInerney P, et al. Scoping Reviews. In: Aromataris E MZE, ed. Joanna Briggs Institute reviewer's manual the Joanna Briggs Institute, 2017. Available via: https://wiki.joannabriggs.org/display/MANUAL/11.1.3+The+scoping+review+framework Accessed 16 February 2020.
42. Peters MDJ, Godfrey CM, Khalil H, et al.: Guidance for conducting systematic scoping reviews. Int J Evid Based Healthc 2015; 13:141-6.
43. World Health Organization (WHO). The universal health coverage (UHC) cube. Health financing for universal coverage and health system performance: concepts and implications for policy. Available via: https://www.who.int/bulletin/volumes/91/8/12-113985/en/.
44. McGowan J, Sampson M, Salzwedel DM, et al.: PRESS Peer Review of Electronic Search Strategies: 2015 Guideline Statement. J Clin Epidemiol 2016; 75:40-6.
45. Tricco AC, Lillie E, Zarin W, et al.: PRISMA Extension for Scoping Reviews (PRISMA-ScR): Checklist and Explanation. Annals of internal medicine 2018; 169:467-73.
46. Levac D, Colquhoun H, O'Brien KK: Scoping studies: advancing the methodology. Implement Sci 2010; 5:69-.

Supplementary files

Appendix 1: Provisional PubMed/MEDLINE search strategy

For peer review only

For peer review only - http://bmjopen.bmj.com/site/about/guidelines.xhtml

Appendix 1: Provisional PubMed/MEDLINE search strategy

Search #	Search Texts and Syntaxes
#1	"implementation science"[Title/Abstract] OR "implementation research"[Title/Abstract] OR "decision science"[Title/Abstract] OR "decision research"[Title/Abstract] OR "improvement science"[Title/Abstract] OR "improvement research"[Title/Abstract] OR "dissemination science"[Title/Abstract] OR "dissemination research"[Title/Abstract]
#2	"programme evaluation"[Title/Abstract] OR "outcome evaluation"[Title/Abstract] OR "process evaluation"[Title/Abstract] OR "impact evaluation"[Title/Abstract] OR "implementation evaluation"[Title/Abstract] OR "implementation fidelity"[Title/Abstract]
#3	facilitators[Title/Abstract] OR barriers[Title/Abstract] OR constraints [Title/Abstract] OR "implementation success"[Title/Abstract] OR implementation failure[Title/Abstract]
#4	#2 OR #3
#5	"Universal health coverage"[Title/Abstract] OR "Universal coverage"[Title/Abstract] OR "population coverage"[Title/Abstract] OR "health equity"[Title/Abstract] OR equity[Title/Abstract] OR equitability[Title/Abstract] OR Health[Title/Abstract] OR "health access"[Title/Abstract] OR "health services"[Title/Abstract] OR "health services accessibility"[Title/Abstract] OR access[Title/Abstract] OR accessibility[Title/Abstract] OR "health insurance"[Title/Abstract] OR "health care insurance"[Title/Abstract] OR "medical insurance"[Title/Abstract] OR "financial risk protection"[Title/Abstract] OR "out of pocket payment"[Title/Abstract] OR "out of pocket expenditure"[Title/Abstract] OR "out of pocket spending"[Title/Abstract]
#6	Africa OR African OR Algeria OR Angola OR Benin OR Botswana OR Burkina Faso OR Burundi OR Cameroon OR "Cape Verde" OR "Central African Republic" OR Chad OR Comoros OR Congo OR "Democratic Republic of Congo" OR Djibouti OR Egypt OR Eritrea OR Eswatini OR Ethiopia OR Gabon OR Gambia OR Ghana OR Guinea OR "Ivory Coast" OR "Cote d'Ivoire" OR Jamahiriya OR Kenya OR Lesotho OR Liberia OR Libya OR Madagascar OR Malawi OR Mali OR Mauritania OR Mauritius OR Mayotte OR Morocco OR Mozambique OR Namibia OR Niger OR Nigeria OR Principe OR Reunion OR Rwanda OR "Sao Tome" OR Senegal OR Seychelles OR "Sierra Leone" OR Somalia OR "St Helena" OR "sub-Saharan

For peer review only

	Africa" OR Sudan OR Swaziland OR Tanzania OR Togo OR Tunisia OR Uganda OR "Western Sahara" OR Zaire OR Zambia OR Zimbabwe
#7	#1 AND #4 AND #5 AND #6

For peer review only

Appendix 1

Preferred Reporting Items for Systematic reviews and Meta-Analyses extension for Scoping Reviews (PRISMA-ScR) Checklist

SECTION	ITEM	PRISMA-ScR CHECKLIST ITEM	REPORTED ON PAGE #
**TITLE**			
Title	1	Identify the report as a scoping review.	1
**ABSTRACT**			
Structured summary	2	Provide a structured summary that includes (as applicable): background, objectives, eligibility criteria, sources of evidence, charting methods, results, and conclusions that relate to the review questions and objectives.	2
**INTRODUCTION**			
Rationale	3	Describe the rationale for the review in the context of what is already known. Explain why the review questions/objectives lend themselves to a scoping review approach.	6-7
Objectives	4	Provide an explicit statement of the questions and objectives being addressed with reference to their key elements (e.g., population or participants, concepts, and context) or other relevant key elements used to conceptualize the review questions and/or objectives.	7
**METHODS**			
Protocol and registration	5	Indicate whether a review protocol exists; state if and where it can be accessed (e.g., a Web address); and if available, provide registration information, including the registration number.	n/a
Eligibility criteria	6	Specify characteristics of the sources of evidence used as eligibility criteria (e.g., years considered, language, and publication status), and provide a rationale.	9-10
Information sources*	7	Describe all information sources in the search (e.g., databases with dates of coverage and contact with authors to identify additional sources), as well as the date the most recent search was executed.	10-11
Search	8	Present the full electronic search strategy for at least 1 database, including any limits used, such that it could be repeated.	Appendix 1
Selection of sources of evidence†	9	State the process for selecting sources of evidence (i.e., screening and eligibility) included in the scoping review.	11
Data charting process‡	10	Describe the methods of charting data from the included sources of evidence (e.g., calibrated forms or forms that have been tested by the team before their use, and whether data charting was done independently or in duplicate) and any processes for obtaining and confirming data from investigators.	12
Data items	11	List and define all variables for which data were sought and any assumptions and simplifications made.	11-12
Critical appraisal of individual sources of evidence§	12	If done, provide a rationale for conducting a critical appraisal of included sources of evidence; describe the methods used and how this information was used in any data synthesis (if appropriate).	n/a

For peer review only

SECTION	ITEM	PRISMA-ScR CHECKLIST ITEM	REPORTED ON PAGE #
Synthesis of results	13	Describe the methods of handling and summarizing the data that were charted.	13
**RESULTS**			
Selection of sources of evidence	14	Give numbers of sources of evidence screened, assessed for eligibility, and included in the review, with reasons for exclusions at each stage, ideally using a flow diagram.	n/a
Characteristics of sources of evidence	15	For each source of evidence, present characteristics for which data were charted and provide the citations.	n/a
Critical appraisal within sources of evidence	16	If done, present data on critical appraisal of included sources of evidence (see item 12).	n/a
Results of individual sources of evidence	17	For each included source of evidence, present the relevant data that were charted that relate to the review questions and objectives.	n/a
Synthesis of results	18	Summarize and/or present the charting results as they relate to the review questions and objectives.	n/a
**DISCUSSION**			
Summary of evidence	19	Summarize the main results (including an overview of concepts, themes, and types of evidence available), link to the review questions and objectives, and consider the relevance to key groups.	n/a
Limitations	20	Discuss the limitations of the scoping review process.	n/a
Conclusions	21	Provide a general interpretation of the results with respect to the review questions and objectives, as well as potential implications and/or next steps.	n/a
**FUNDING**			
Funding	22	Describe sources of funding for the included sources of evidence, as well as sources of funding for the scoping review. Describe the role of the funders of the scoping review.	14

JBI = Joanna Briggs Institute; PRISMA-ScR = Preferred Reporting Items for Systematic reviews and Meta-Analyses extension for Scoping Reviews.

* Where *sources of evidence* (see second footnote) are compiled from, such as bibliographic databases, social media platforms, and Web sites.

† A more inclusive/heterogeneous term used to account for the different types of evidence or data sources (e.g., quantitative and/or qualitative research, expert opinion, and policy documents) that may be eligible in a scoping review as opposed to only studies. This is not to be confused with *information sources* (see first footnote).

‡ The frameworks by Arksey and O'Malley (6) and Levac and colleagues (7) and the JBI guidance (4, 5) refer to the process of data extraction in a scoping review as data charting.

§ The process of systematically examining research evidence to assess its validity, results, and relevance before using it to inform a decision. This term is used for items 12 and 19 instead of "risk of bias" (which is more applicable to systematic reviews of interventions) to include and acknowledge the various sources of evidence that may be used in a scoping review (e.g., quantitative and/or qualitative research, expert opinion, and policy document).

Adapted from: Tricco AC, Lillie E, Zarin W, O'Brien KK, Colquhoun H, Levac D, et al. PRISMA Extension for Scoping Reviews (PRISMAScR): Checklist and Explanation. Ann Intern Med. 2018;169:467–473. doi: 10.7326/M18-0850.

For peer review only - http://bmjopen.bmj.com/site/about/guidelines.xhtml
